# Psychometric properties of the Czech moral injury symptom scale

**DOI:** 10.1038/s41598-025-97530-1

**Published:** 2025-04-15

**Authors:** Karolina Vlckova, Miroslava Janouskova, Lucie Bankovska-Motlova

**Affiliations:** 1 Third Faculty of Medicine, Charles University, Prague, Czech Republic; 2 Thomayer Faculty Hospital, Prague, Czech Republic

**Keywords:** Moral injury, Healthcare professionals, Covid-19, MISS-HP, Human behaviour, Health care

## Abstract

Moral injury is defined as a deep sense of transgression, including feelings of shame, grief, meaninglessness, and remorse from having violated core moral beliefs. This study aimed to adapt the Moral Injury Symptom Scale-Healthcare Professionals (MISS-HP) for measure this concept in the Czech Republic and explore its psychometric properties. Sample of healthcare providers (N = 694) completed the MISS-HP questionnaires, the Shirom Melamed Burnout Measure (SMBM) and the Professional Quality of Life scale (PROQoL). Cronbach´s alpha of MISS-HP was found to be 0.62; exploratory factor analysis returned 4 factors. Correlations with SMBM and PROQoL were moderate (from 0.31–0.46), which confirmed the construct validity of MISS-HP. ROC curve analysis identified the optimal cut-off score at 42 points with 75% sensitivity and 69% specificity. Female gender, younger age and atheism were related to higher symptoms of moral injury. Czech MISS-HP was found to be a valid and reliable measure of moral injury.

## Introduction

Moral injury (MI), originally discussed with transgressing moral beliefs and values during wartime among military personnel, has expanded beyond this context to include similar emotions experienced by healthcare professionals, first responders, and others experiencing moral emotions resulting from actions taken or observations made during traumatic events or circumstances^1^. MI is defined as a deep sense of transgression including feelings of shame, grief, meaninglessness, and remorse from having violated core moral beliefs^2^. This concept is distinct from moral distress, which includes emotions such as powerlessness, frustration/anger and sadness^3^. MI then is associated with perpetration, failure to prevent, or witnessing morally-transgressive acts^4^. Moral distress is related more to situational problems, while MI represents an experience of the problem that results in a long-lasting change to an individual’s sense of losing hope, trust, or integrity, including malfunctioning^5^. Long-lasting feelings of guilt can have a serious impact on the mental state of healthcare professionals, resulting in developing post-traumatic stress disorder (PTSD), suicidal ideation or substance abuse^5,6^. The Covid-19 pandemic promoted interest in the psychological strain experienced by exhausted healthcare professionals^7^ who were expected to solve dilemmas (e.g. decision-making about hospitalization or even the placement of a patient on a ventilator when there was a shortage of resources, or being allocated to take care of patients with a condition for which they were not qualified, or not having enough resources for patients with diseases other than Covid, etc.) A qualitative multi-centre study from Italy confirmed our clinical experience that the pandemic was associated with stressors caused by limited healthcare resources, intensified patient triage, changeable selection criteria, limited therapeutic/clinical knowledge, and patient isolation. Healthcare professionals also felt isolated from non-healthcare professionals, alienated from patients, and betrayed by colleagues, administrators and the public^8^. Another source of stress was that healthcare professionals experienced much more death of their patients than they had to face during non-pandemic situations^9^. A cross-sectional study with physicians and nurses using the Moral Injury Symptom Scale (MISS-HP) showed positive correlations of MI with depression, anxiety, low well-being and burnout symptoms^10^. The prevalence of MI was 41% in that sample, suggesting that it is a serious problem that needs our attention^10^. Searching for possible causes of mental health problems in physicians (such as undiagnosed MIs) becomes more important as the level of burnout increases^11^ and the suicide rate in this population is higher than in the general population^12,13^. Workplace burnout has been recognised in the ICD-11 as a diagnosable condition (diagnostic code QD85), “resulting from chronic workplace stress and encompassing a constellation of exhaustion, cynicism and reduced efficacy”^14^. However, there is an overlap in its conceptual understanding: the signs and symptoms that warrant burnout syndrome and MI suggest that MI should be included in any discussion of physician burnout^15^. Therefore, more research in this field is also needed to distinguish between moral distress, MI and professional burnout, thus allowing us to tailor the treatment. In order to recognize MI in healthcare and subsequently provide adequate treatment measures, Koenig et al.^16^ developed the 10-item Moral Injury Symptoms Scale—Health Professional (MISS-HP).

The purpose of this study is to adapt MISS-HP to the Czech Republic and assess its psychometric properties among healthcare professionals.

## Methods

This was a brief cross-sectional online survey among healthcare professionals. Participants were selected by snowball technique and convenience sampling using dissemination through relevant healthcare professional organisations and personal contacts in order to get heterogenous sample. The minimum required sample size (N = 222) was calculated based on the expected Cronbach alpha (0.75), the minimum required Cronbach alpha of 0.65, the expected dropout rate (30%) and the number of items in the questionnaire (11)^17^. Data were collected from March to June 2022. Inclusion criteria were: being both clinical and non-clinical healthcare staff, working in healthcare for at least 2 years. Exclusion criteria were: having a work break of longer than 6 months in the last 2 years. Participants filled out informed consent and completed 3 questionnaires—MISSHP, the Professional Quality of Life scale, the Shirom Melamed Burnout measure and demographic variables. The study is reported based on the COSMIN checklist^18^.

### Czech version of the moral injury symptom scale (MISS-HP)

MISS-HP is a 10-item questionnaire that assesses multiple dimensions of MI as suggested by experts in this field^16^. It is designed for screening MI in healthcare professionals^16^. The 10 dimensions of MI assessed by this measurement are betrayal, guilt, shame, moral concerns, loss of trust, loss of meaning, difficulty forgiving, self-condemnation, religious struggle and loss of religious faith^16^. For every item, there is a visual analogue scale ranging from 1 to 10. After recoding the positively worded items (5, 6, 7, 8 and 11), item scores are added up to create a total score ranging from 10 to 100, with higher scores indicating greater MI.

The Czech version of MISS-HP was developed according to a guideline^19^. Translation into Czech was done by forward and backward translation by independent translators, with the final version being discussed by the research team. This version was assessed with 5 physicians using cognitive reviews with semi-structured interviews^20^. Participants were asked about appropriateness and clarity of questions and responses. After 5 interviews this process was stopped due to data saturation^21^. Its content validity was confirmed; however, it was suggested to split the question about unforgiveness, as it asks about two different aspects – whether healthcare providers forgive themselves for how they treat their patients and whether they forgive themselves for the consequences of their actions/behaviour. Adding this new item, the total score ranges from 11 to 110, with higher scores indicating greater MI. Immediately following the completion of the MISS-HP, participants were asked the following: “Do the feelings you indicated above cause you significant distress or impair your ability to function in relationships, at work, or other areas of life important to you? In other words, if you indicated any problems above, how difficult have these problems made it for you to do your work, take care of things at home, or get along with other people?” There were 5 response options: “not at all,” “mild,” “moderate,” “very much,” and “extremely.” This question was added similarly as in original study^16^ in order to get criteria for functional disability.

### Professional quality of life scale (PROQOL)

This is a 30-item scale which has the goal of measuring the professional quality of life, particularly the quality one feels toward his or her work as a helper^22^. The scale has 3 dimensions—a compassion satisfaction subscale, a burnout syndrome subscale, and a secondary traumatic subscale^22^. Compassion satisfaction means having pleasure from work; a higher score on this scale means greater satisfaction. Burnout syndrome and secondary trauma are part of compassion fatigue. The burnout subscale is related to feelings of hopelessness and difficulties dealing with work. The secondary traumatic subscale concerns stressful work-related events^22^. This scale has not been validated in the Czech Republic yet; therefore, we did a backward translation and cognitive interviews to be able to use it in this study, as there is no other gold measure besides the burnout syndrome measure that we could use for the validation of MISS-HP.

### The Czech version of the shirom melamed burnout measure (SBMB)

The SMBM is quite a new measurement for assessing burnout symptoms; however, it was proven to be reliable, valid and stable^23^. The measurement consists of the three subscales labelled physical fatigue (six items: e.g. “I feel like my batteries are dead.”); cognitive weariness (five items: e.g. “I feel I am not thinking clearly.”); and emotional exhaustion (three items: e.g. “I feel I am unable to be sensitive to the needs of co-workers or clients.”) The items have a visual analogue scale ranging from 1 (never or almost never) to 7 (always or almost always)^24^. Higher scores indicate a higher level of burnout syndrome. Czech standardization and psychometric properties are available^25^. The study was performed in line with the principles of Helsinki declaration and it was approved on 24 February 2022 by the Ethical Committee of the Third Faculty of Medicine at Charles University (Ref: MOI-01-2022). All participants gave informed consent at the beginning of the online survey.

### Statistical analysis: demographic variables

We assessed the association between demographic variables and scores in MISS-HP using Pearson correlation for continuous variables and Student’s t-test or ANOVA for categorical variables. Stepwise linear regression was used to determine the association between dependent variables (scores in MISS-HP) and independent variables.

### Item analysis

For every item of MISS-HP, we computed the mean and standard deviation. We also computed Item difficulty, which was calculated using item mean and converted to interval < 0; 1 > using a formula mean-scale min/(scale max-scale min). Correlations with total score without a particular item were also provided.

### Reliability

Reliability was assessed with internal consistency using Cronbach´s α for a total score of MIS. Realiability of questionnaire was also supported using cluster analysis which was carried out in two subsequent steps, hierarchical and k-means clustering techniques, in order to find the optimal number of clusters.

### Exploratory factor analysis

Exploratory factor analysis (EFA) using principal components factor analysis (PCA) with Promax rotation was conducted on the MISS-HP items using the Kaiser-Guttman rule, which states that the number of factors to be extracted should be equal to the number of factors having an eigenvalue of greater than 1.0.

### Validity

To determine whether the MISS-HP scale measures new constructs than those that already exist, we used the Pearson correlation between the MISS-HP and religiosity; we expected a low or zero correlation. Construct validity was assessed using Pearson correlation among MISS-HP, SBMB, and PROQoL; we expected a moderate correlation (above 0.5).

### Cut-off determination

To determine the best cut-off on the MISS-HP that could be used to identify clinically significant MI, a receiver operator curve (ROC) analysis was performed. The cut-off on the MISS-HP was determined based on the total score that was most sensitive and specific for identifying clinically significant functional disability by applying Youden’s index. The positive predictive value and negative predictive value for the cut-off scores were also determined.

All missing values were excluded from the analysis. A significant p-value was set at 0.05. All analyses were conducted within SPSS version 28.01.

## Results

### Sample

There was a total sample of 1678 healthcare professionals at the start of the survey and 694 of them completed the whole process (41% completion rate). The survey was online from March to June 2022. Demographic information is presented in Table 1; the mean age was 44.8 (standard deviation = 11.5). Other professions who completed the survey were psychologists, social workers, nurse assistants, and physiotherapists. Females and younger people scored higher in MISS-HP. Religious people and professionals in outpatient settings scored lower. The length of practice was negatively correlated with the score in MISS-HP, meaning that healthcare professionals with shorter careers scored higher. Multivariate stepwise regression (R = 0.262) showed that only sex, age and religiosity are significant predictors for the score in MISS-HP; the strongest predictor was religiosity (see Table 2).

**Table 1:** Demographics.

Characteristic	%	n
Sex		
Female	86.5	600
Male	13.5	94
Age		
20–34	21	143
35–49	46	319
50–64	28	196
65 +	5	36
Religiosity		
Yes	35	246
No	65	448
Profession		
Physician	36	250
Nurse	50	349
Other	14	95
Work area		
Inpatient clinic	68	474
Outpatient clinic	17	121
Other	15	99

**Table 2:** Regression analysis.

Characteristic	Mean MI (SD)	Stepwise Multivariate Regression β (SE)
Sex		
Female	40.0 (12.1)**	0.116 (3.108)**
Male	35.2 (11.3)	
Age		
20–34	42.6 (12.7)**	− 0.141 (-3.782)**
35–49	38.9 (11.5)	
50–64	38.9 (12.7)	
65 +	33.7 (10.5)	
Religiosity		
Yes	36.4 (12.3)**	0.176 (4.782)**
No	41.0 (11.8)	
Profession		
Physician	37.4 (12.4)**	
Nurse	41.2 (11.7)	
Other	38.0 (12.5)	
Work area		
Inpatient clinic	40.3 (12.2)*	
Outpatient clinic	36.1 (11.7)	
Other	38.9 (11.9)	
Length of practice	M = 21.3 (12.2)	− 0.1**

SD means standard deviation Pearson correlation (r) was used to determine the association between MISS-HP and continuous variables. Student’s t-test or ANOVA was used to compare average MISS-HP scores across categorical variables. Significant results are in bold (*p < .05. **p < .001).

In Table 3, we present descriptive statistics of all MISS-HP items. We also evaluated each item’s difficulty and correlation with the total score. The minimum difficulty was 0.1 (Self-condemnation and Feeling punished by God); the maximum was 0.6 (Loss of faith). Most of the item-total correlations were higher than 0.2; only for the Loss of Faith item, the total correlation was 0.01. The mean score on the scale was M = 39. 36 (standard deviation = 12.18); the range score was from 12 to 76.

**Table 3:** Distribution of scores and item analysis.

Item	M	SD	Item difficulty	Item-total correlation
1. Betrayal	4.5	2.8	0.4	0.3
2. Guilt	3.0	2.6	0.2	0.3
3. Shame	2.9	2.4	0.2	0.4
4. Moral concerns	3.2	2.6	0.2	0.4
5. Loss of trust	4.4	2.5	0.4	0.2
6. Loss of meaning	2.7	2.1	0.2	0.2
7. Unforgiveness to myself	4.2	2.5	0.4	0.4
8. Unforgiveness	4.2	2.6	0.4	0.3
9. Self-condemnation	1.8	1.7	0.1	0.4
10. Feeling punished by God	1.6	1.5	0.1	0.3
11. Loss of religious faith	6.8	3.0	0.6	0.01

M = mean; SD = standard deviation.

**Fig. 1:**
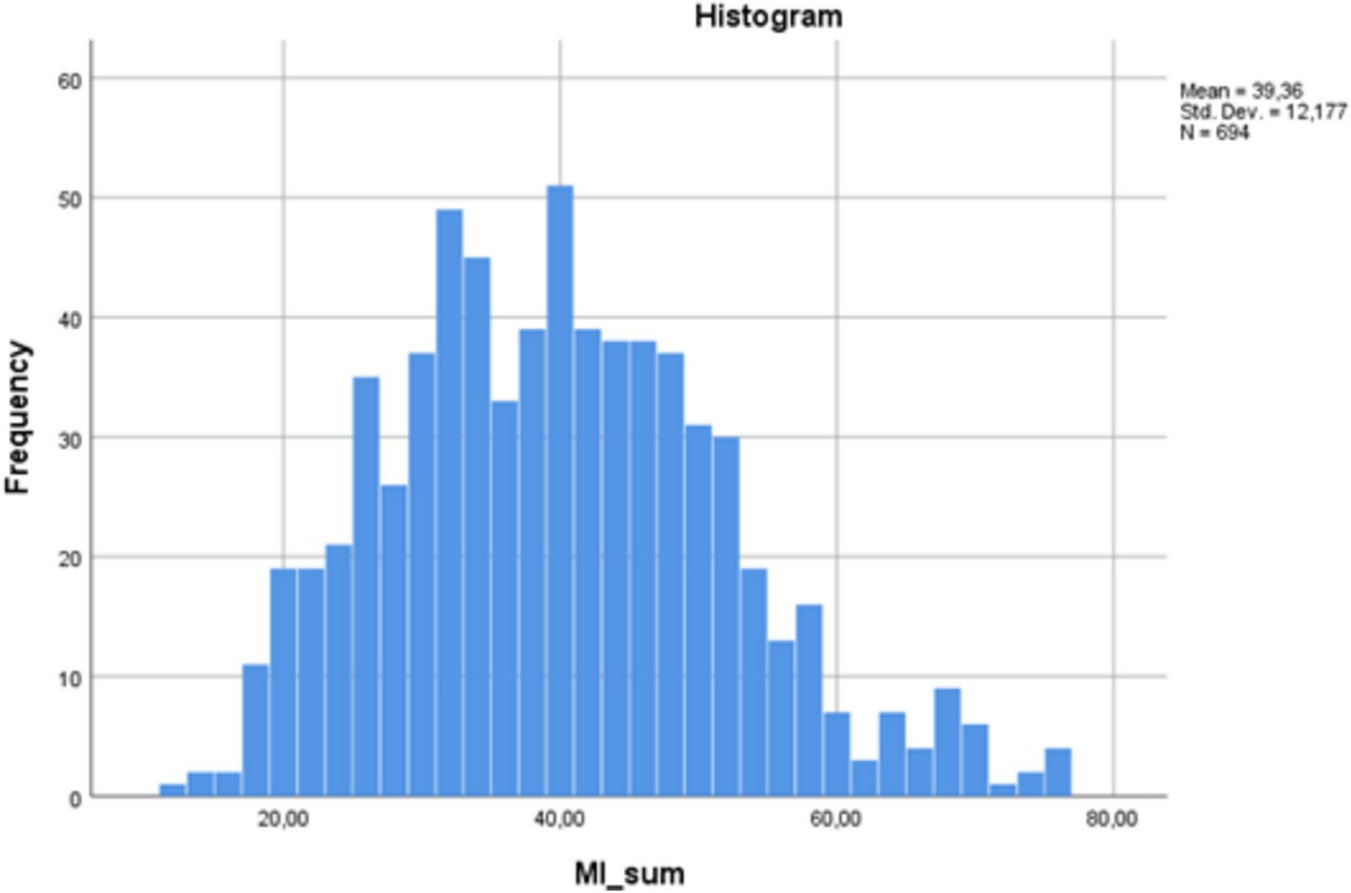
Histogram of sum scores of MISS-HP.

Scores are presented in Fig. 1; 17% of the sample confirmed having serious problems at work or home related to MI.

### Reliability

The Cronbach´s alpha for the total score of 11 items was 0.622.

### Cluster analysis

Cluster analysis (Table 4) found two major clusters (235 individuals belonging to the first cluster and 459 subjects to the second). These two clusters approximately divided the sample into a group of individuals with severe symptoms of moral injury (M = 58.3) and a group with less severe or no symptoms of moral injury (M = 45.1). ANOVA results indicated significant differences between the cluster for all variables and 24.207 was the final distance between the two clusters.

**Table 4:** Cluster analysis.

MISS-HP	Cluster				Error		F	*p*-value
Item	1	2	Mean square	DF	Mean square	DF		
1. Betrayal	6	4	893.962	1	6.737	687	132.699	< 0.001
2. Guilt	5	2	1113.602	1	4.895	687	227.478	< 0.001
3. Shame	5	2	2055.140	1	2.566	687	800.927	< 0.001
4. Moral concerns	6	2	2895.019	1	2.493	687	1161.271	< 0.001
5. Loss of trust	6	7	90.728	1	6.274	687	14.460	< 0.001
6. Loss of meaning	8	8	47.035	1	4.323	687	10.880	< 0.001
7. Unforgiveness to myself	6	7	77.894	1	6.143	687	12.679	< 0.001
8. Unforgiveness	6	7	30.778	1	6.693	687	4.599	< 0.001
9. Self-condemnation	3	1	226.536	1	2.444	687	92.678	< 0.001
10. Feeling punished by God	2	1	102.373	1	2.183	687	46.886	< 0.001
11. Loss of religious faith	5	4	58.518	1	8.909	687	6.569	< 0.001

Note: DF = degree of freedom.

### Exploratory factor analysis

Exploratory factor analysis with Promax rotation returned a 4-factor model, which explains the 61% variance (see Table 5). The Kaiser-Meier test (KMO 0.657) and Bartlett’s test of Sphericity (chi square 1544.9, df 55; *p* < 0.001) confirmed the use of exploratory factor analysis. Factor 1 consisted of 5 items (M2, M3, M4, M9) and is related to the feelings of failure. Factor 2 has 2 items (M7, M8) and covers forgiveness. Factor 3 covered 2 items (M1, M5) that are related to trust in colleagues. Factor 4 consisted of 2 items (M6, M11) that are related to searching for meaning in work.

**Table 5:** Exploratory factor analysis.

Item	Factor 1	Factor 2	Factor 3	Factor 4
1. Betrayal	0.280	0.122	**0.750**	**− **0.181
2. Guilt	**0.691**	0.066	0.054	**− **0.091
3. Shame	**0.825**	0.003	0.290	**− **0.103
4. Moral concerns	**0.743**	0.001	0.383	**− **0.100
5. Loss of trust	0.018	0.043	**0.791**	0.278
6. Loss of meaning	0.140	0.140	0.283	**0.758**
7. Unforgiveness to myself	0.146	**0.916**	0.083	0.218
8. Unforgiveness	0.078	**0.912**	0.091	0.191
9. Self-condemnation	**0.635**	0.120	0.046	0.272
10. Feeling punished by God	**0.511**	0.229	-0.036	0.063
11. Loss of religious faith	-0.114	0.191	-0.167	**0.633**
**Initial eigenvalues**	**24.2**	**16.4**	**11.6**	**9.3**

Note: The highest eigenvalues are in bold.

### Discriminant validity

Correlation with religiosity was 0.18 (significant at the *p* > 0.001 level).

### Construct validity

Table 6 presents the correlation of the SBMS scale and PROQoL with MISS-HP. The correlation of those instruments with MISS-HP is statistically significant at a level of 0.01, which is moderate.

**Table 6:** Correlation of MISS-HP with PROQoL and SBMB Scales.

Measure and Dimension	M (SD)	Pearson Correlation with MISS-HP
PROQoL		
PROQoL Compassion Satisfaction Subscale	38.2 (6.0)	**− **0.31**
PROQoL Burnout Syndrome Subscale	21.4 (5.9)	0.46**
PROQoL Secondary Traumatic Scale	22.7 (5.8)	0.37**
SBMB		
SBMB Physical Domain	22 (7.6)	0.40**
SBMB Cognitive Domain	14.5 (5.9)	0.37**
SBMB Emotional Domain	8.3 (3.4)	0.34**
SBMB Sum	45 (14.6)	0.44**

SD = standard deviation. **p < .001.

### Cut-off determination

**Fig. 2:**
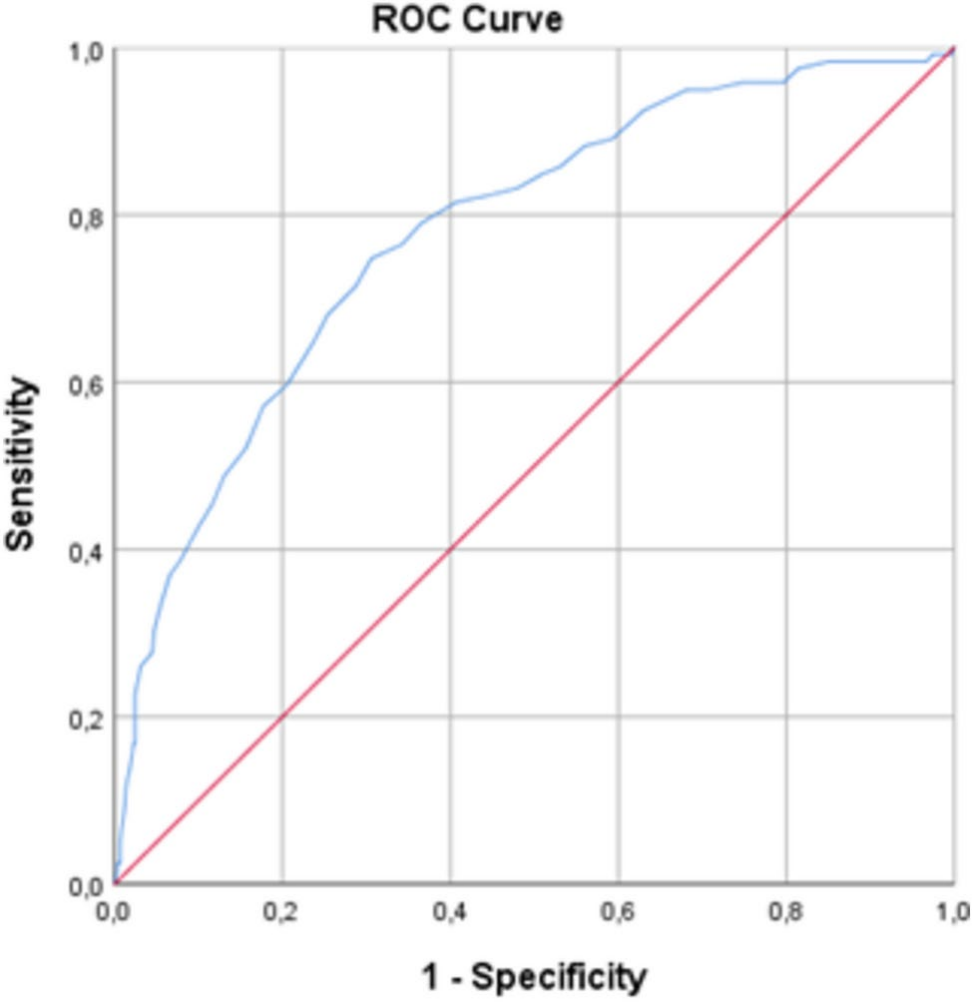
ROC curve.

The area under the curve (Fig. 2) is 0.779 (asymptotic 95% CI = 0.773–0.825); the standard error is 0.023. Applying Youden’s index (sensitivity + specificity—1), we identified the optimal cut-off score as 42 points or higher. With such a score, MISS-HP has a sensitivity of 75% and a specificity for detecting significant functional impairment at 69%. The positive predictive value was 32% (of the 288 with positive tests, 91 were impaired). The negative predictive value was 93% (of the 406 without impairment, 378 had a negative test).

## Discussion

We showed that the Czech version of MISS-HP is a valid and reliable measure with good psychometric properties that can be used in the Czech Republic. Item analysis showed that almost all of the items of MISS-HP meet the requirements for item difficulty and item-total correlation. The exception was the item Loss of Faith, which did not correlate with the sum score in the MISS-HP. Splitting the question about unforgiveness, which was suggested by physicians in the cognitive interviews, seemed reasonable, and item analysis confirmed that those questions are both important. In a previous validation study in Germany providing a report of the item Loss of Faith, it was also found to have insufficient psychometric properties and was suggested to be left as optional for international comparison^26^. Reliability measured by alpha was found to be sufficient, although a little bit lower than in previous studies^16,26^, which could be explained by the multi-dimensionality of this scale. These findings were also corroborate by cluster analysis. However, also other aspects of the reliability needs to be assessed, e.g. the temporal stability of this scale, which has not yet been done in any study.

An analysis of the influence of demographic variables on scores in MISS-HP showed that women and younger people scored higher in MISS-HP. People who do not consider themselves religious and those working with in-patients also scored higher. The shorter length of practice is correlated with a higher score in MISS-HP. However, multivariate regression confirmed sex, age and religiosity as significant predictors. Age and female gender as significant predictors were also confirmed in other studies^10,27^. Contrary to our findings, a study from China found that Buddhist/Taoist religious beliefs were also related to a higher MI score, which indicates that this concept seems to be a culturally sensitive phenomenon. In our study, the relationship to religion was found with the opposite association, which may be explained by the fact that the Czech Republic is among the least religious countries in Europe^28^. Contrary to our results and previous studies, in the German sample, demographic variables were not found to play a role^26^. Previous studies also showed that anxiety, depression and medical errors in the previous month were also significant predictors^27^. A previous study showed stronger correlation between burnout and MI^27^; however, this study used a different measure for burnout syndrome. In our study, the correlation with burnout syndrome was lower than expected, indicating that MI is a different concept than burnout syndrome. The strongest correlation was with the physical domain of burnout syndrome. Correlation with domains in PROQoL had similar strength; the strongest correlation of MISS-HP was with the burnout syndrome subscale, thus a negative association with the compassion satisfaction subscale is reasonable. We also used the correlation of MISS-HP and religiosity as a demonstration of the discriminant validity of this measurement, as in a previous study^16^. The exploratory factor analysis showed 4-factor structures in our sample, which is different from previous studies^10,16,26^, probably capturing the cultural specificity of this phenomenon.

Cut-off was determined at 42 points, which is higher than in previous studies^16,26^ but lower than in one study^10^; however, in our study, the range of scores is also higher as we added one question, contrary to colleagues from Germany who did not use the item Loss of faith in their final score^26^. Our specificity and sensitivity were similar to those found in the Chinese study^10^. Applying a cut-off score on our sample, the prevalence of MI would be 13%; previous studies showed results that varied from 7 to 20%^10,16^. However, we did not aim at having a representative sample; therefore, we cannot confirm how many healthcare providers suffer from that. Those studies, on the other hand, showed that it is an important distinct phenomenon and has an influence on the quality of life, as well as on depression^10,16^, thus it needs our attention. Our cut-off scores showed 75% sensitivity and 69% specificity, which are considered sufficient for screening instruments. Cut-off determination should be also verified using different way for assessing functional disability or any external criteria.

As many studies before, this study also showed that burnout syndrome and MI are related concepts; however, distinctions do exist. Therefore, the interventions for preventing or treating these issues must be different. There is a need for increased education about this phenomenon among healthcare professionals who are at risk (especially the young and females) and supporting them in seeking informal or formal help as quickly as possible^29^. Moreover, there is a need for the development and evaluation of interventions specifically designed for MI.

There are several limitations in our study. Our completion rate was very low, which is typical for online surveys. One of the limitations of our study is also using the snowball method. This method may have potential bias but we aimed to adapt this method in Czech, not to provide a representative sample, therefore we use it as a pragmatic method which allows us to get access to as many healthcare providers as possible in a short period. Moreover, it was found a good way how to reach healthcare providers for sensitive topics^30^. The convenience sample could cause a non-respond bias and the absence of some of the data which may be overcome in future studies using neural networks^31^. However, the sample is robust enough for the Czech Republic and exceeded the required file size calculated by power analysis. On the other hand, generalizability is limited as we did not have representative samples related to age, gender or profession. This was not the aim of this study; we aimed to adapt the MISS-HP to the Czech context, which was achieved. This reliable method could be used in future research for a representative study that could reveal the prevalence of this phenomenon, which is highly needed.

## Conclusion

This study showed that the Czech version of MISS-HP is a reliable and valid measurement. The scale should be used for screening MI among healthcare professionals to identify those with clinical manifestations and support them in seeking informal or formal help as quickly as possible. We found that younger, atheist and female healthcare professionals are at higher risk for developing MI. There is a need for a prevalence study among healthcare professionals, and the development of educational programs and specific interventions to help those with clinical symptoms. Adequate prevention should be provided, with education about MI being the first step. It was also found that moral injury is a very culturally sensitive phenomenon, therefore prevention, education and interventions also need to be culturally sensitive.

## Data Availability

Data from this study are available upon reasonable request please contact karolina.vlckova@lf3.cuni.cz to get them.
